# Efficacy and safety of subcutaneous interferon-β-1a in patients with a first demyelinating event and early multiple sclerosis

**DOI:** 10.1517/14712598.2014.924496

**Published:** 2014-06-26

**Authors:** Mark S Freedman

**Affiliations:** ^a^ University of Ottawa and the Ottawa Hospital Research Institute, Department of Medicine, 501 Smyth Road, Ottawa, Ontario, K1H 8L6, Canada+1 613 737 8532; +1 613 737 8162; mfreedman@toh.on.ca

**Keywords:** first clinical demyelinating event, inflammation, multiple sclerosis, subcutaneous IFN-β-1a

## Abstract

***Introduction:*** Multiple sclerosis (MS) is an inflammatory demyelinating disease of the CNS. Evidence suggests that MS should be treated as early as possible in order to maximize the benefit of treatment.

***Areas covered:*** This review details current understanding about the treatment of relapsing–remitting MS (RRMS). The pharmacological and clinical data on the use of subcutaneous (s.c.) interferon β-1a (IFN-β-1a) as a therapeutic option for RRMS are covered, with a focus on the importance of treating patients with MS as early as possible in the course of the disease, in order to delay permanent axonal damage that is responsible for the signs and symptoms of disease progression.

***Expert opinion:*** There is a wealth of data on the treatment of RRMS with s.c. IFN-β-1a indicating that patients treated during the early inflammatory stages of the disease have significantly improved short-term outcomes compared with patients who commence treatment late. It remains to be determined whether the short-term effects of early treatment will translate into long-lasting benefits, although it is hoped that ongoing research will help to answer this question.

## 1. Background

### 1.1 Relapsing–remitting multiple sclerosis

Multiple sclerosis (MS) is an inflammatory demyelinating disease of the CNS that can lead to severe disability. In most patients, MS is characterized by recurrent attacks of neurological dysfunction consistent with demyelination and axonal damage within the CNS, followed by periods of partial or complete remission and recovery – a pattern of MS known as relapsing–remitting MS (RRMS) [1].

The limited clinical symptoms and the presence of partial or complete remission of function during the early stages of RRMS can be explained by successful remyelination and neuroplasticity, which involves some reorganization of brain function that can compensate and mask early brain damage [2]. It is this balance between damage and repair that dictates disease progression. Indeed, there are often many more lesions present in the CNS than there are symptoms of MS during the early stages of the disease. However, in some cases, significant disability can occur in the presence of a small number of lesions if they are located in areas such as the brainstem and spinal cord [3]. Eventually, remyelination and repair mechanisms can no longer compensate for the lost tissue and permanent loss of function occurs. At this stage of the disease, which is likely to be the transitional period to secondary progressive MS (SPMS), there is a greater chance that any new lesion will lead to neurological disability. Consequently, early treatment will aid ongoing repair mechanisms by limiting damage. However, whether early treatment provides long-term benefits is yet to be determined – it is hoped that ongoing research will answer this question.

Neurological dysfunction is caused by inflammation that results in damage to myelin. This damage is evident as cerebral white matter lesions that can be detected by MRI. Although there is a predilection for white matter, gray matter disease is also prominent and is often the cause of cognitive decline [4]. The number of brain lesions present at the time of the first clinical manifestation of MS is prognostic of early relapses and disability [3], although these lesions are frequently clinically silent. Evidence suggests that this underlying pathology is present from the start of the disease, sometimes long before clinical signs are present, and continually progresses during the course of the disease [5,6]; hence, some patients can accumulate significant silent damage before symptoms of MS appear, whereas others present very early in the course of the disease with only a few lesions. This is similar to other conditions that often develop silently before symptoms develop; when presenting early, they dictate a different therapeutic approach than when they present late (e.g., coronary disease and cancer).

### 1.2 Pharmacodynamics of injectable therapies

Well-established first-line injectable drugs for MS include subcutaneous (s.c.) IFN-β-1a, intramuscular (i.m.) IFN-β-1a, s.c. IFN-β-1b and glatiramer acetate (GA). These drugs are most effective in the early inflammatory phase of MS [7-10]. Their precise mechanisms of action are not fully understood, but they are believed to work by modifying the inflammatory response within the CNS, preventing damage from accumulating [11,12].

### 1.3 Diagnosing MS

The growing need for early diagnosis and treatment of MS has led to significant evolvement of the MS diagnostic criteria [13-16]. The first diagnostic criteria (commonly known as the Poser criteria) were published in 1983 [13]. These criteria allowed a diagnosis of MS after the occurrence of a second clinical attack at least 1 month after the first attack, with evidence of white matter involvement in more than one site in the CNS. However, as it can take many years for some patients to experience a second attack, these criteria resulted in a delay in diagnosis and treatment while subclinical damage was occurring, and, even today, some physicians still use these criteria.

The McDonald criteria were published in 2001 [14] and were updated in 2005 and 2010 [15,16]. These criteria introduced MRI evidence of the disease [14], allowing subclinical disease – which may develop in the absence of any further clinical symptoms – to form part of the diagnostic algorithm. As such, a second clinical event was no longer required for a diagnosis of MS. In patients with symptoms suggestive of MS, MRI is an important diagnostic tool as white matter lesions are a strong predictor of MS relapses, as well as having a strong correlation with long-term disability [17]. Each revision of the McDonald criteria has allowed diagnosis to be made sooner, that is, after the first attack.

For some physicians, a definite diagnosis of MS is a requirement for initiating therapy, whereas others initiate therapy following an event suggestive of MS and before patients fulfill the diagnostic criteria. The McDonald criteria are diagnostic criteria and not treatment criteria; however, prophylactic treatment is warranted, just as it would be for a patient presenting with a transient ischemic attack in order to reduce the chances of a future stroke.

### 1.4 First clinical demyelinating event and evidence for early MS treatment

The first manifestation of MS is usually a single acute first clinical demyelinating event (FCDE), attributable to selective damage within the CNS [2], although there is frequently MRI evidence – sometimes extensive – of prior ‘silent’ damage. It has been estimated that as many as 70% of patients who present with an FCDE will eventually experience a second attack [3]; however, this number may be higher when clinical and MRI parameters are taken into consideration (2010 McDonald criteria). Further, a recent review of trials carried out in patients with an FCDE suggests that 85% of these patients will meet the 2010 McDonald MS criteria within 2 years [18].

Trials exploring the effects of GA and IFN-β in patients with an FCDE suggestive of MS have shown that early treatment delays evidence of new disease activity and that it is advantageous to treat immediately after the first clinical signs of MS manifest [7-10]. Recently, the Rebif Flexible Dosing in Early MS (REFLEX) study and its ongoing preplanned extension, Rebif Flexible Dosing in Early MS Extension (REFLEXION), demonstrated the benefits of early intervention with s.c. IFN-β-1a [19-21]. Herein, we will review the data supporting the use of s.c. IFN-β-1a in the early treatment of MS (Box 1).

**Box 1.** **Drug summary.**

Drug name	Rebif®, Merck Serono Europe Ltd
Phase	Approved
Indication	Relapsing multiple sclerosis (MS)/first clinical demyelinating event suggestive of MS
Route of administration	Injectable
Chemical structure	Biological, protein, recombinant
Chemical name	Interferon β-1a

Pharmaprojects – copyright to Citeline Drug Intelligence (an Informa business). Readers are referred to Citeline (http://informa.citeline.com).

## 2. Clinical efficacy

### 2.1 Efficacy of sc IFN-β-1a in MS

The Prevention of Relapses and Disability by IFN-β-1a Subcutaneously in Multiple Sclerosis (PRISMS) study established the efficacy of s.c. IFN-β-1a (44 or 22 µg) three times a week (t.i.w.) versus placebo over 2 years, followed by a 3- and 4-year extension [22,23]. Patients enrolled were diagnosed as having clinically definite MS (CDMS) or laboratory-supported definite MS of at least 1 year’s duration with an Expanded Disability Status Scale (EDSS) score of ≤ 5.0 and at least two relapses in the proceeding 2 years [22]. Patients were diagnosed with MS based on the Poser criteria; therefore, many patients included in PRISMS did not receive a diagnosis for up to 5 years after the FCDE, compared with patients today who are usually diagnosed using the McDonald criteria. As such, some of the patients in PRISMS receiving placebo did not receive treatment for ≥ 7 years [22].

Patients receiving s.c. IFN-β-1a had significantly fewer relapses over 2 years compared with placebo (mean number of relapses per patient: 1.73 for 44 µg; 1.82 for 22 µg; 2.56 for placebo; p < 0.005 for each active group versus placebo) and prolonged (median) delay compared with placebo of first on-study relapse (5 months for 44 µg; 3 months for 22 µg) [22]. A higher proportion of patients were relapse-free at 2 years (32% for 44 µg, p < 0.005; 27% for 22 µg, p ≤ 0.05; 16% for placebo), and a delay in disability progression (first quartile) was observed (21.3 months for 44 µg; 18.5 months for 22 µg; 11.9 months for placebo; p < 0.05 for each active group versus placebo). Change in EDSS score from baseline and the number of T2 active lesions on MRI over 2 years were also significantly lower in both s.c. IFN-β-1a groups, compared with placebo. The burden of disease measured with proton density T2 MRI also demonstrated a median decrease (3.8% for 44 µg and 1.2% for 22 µg), compared with a median increase of 10.9% in placebo-treated patients [22]. After 2 years, patients who had initially received placebo crossed over to s.c. IFN-β-1a (44 or 22 µg; delayed-treatment [DT] group) and patients receiving the study drug continued on their original regimen for a further 2 years [23]. At 4 years, patients consistently treated with both doses of s.c. IFN-β-1a had significantly better outcomes, compared with those who initially received placebo, in terms of proportion of patients free from relapse (19.0% for 44 µg; 14.4% for 22 µg; 6.7% for DT) and time to second relapse (16.9 and 8.3 months longer for 44 and 22 µg, respectively, compared with DT). Time to first confirmed EDSS score progression (42.1 months for 44 µg; 24.2 months for DT; p = 0.047) and number of new T2 lesions (p < 0.001) were also better at 4 years in patients consistently receiving s.c. IFN-β-1a [22], supporting the rationale that early intervention provides benefits over DT.

A follow-up of PRISMS scheduled 7 – 8 years after study start has demonstrated the long-term efficacy of patients initially randomized to s.c. IFN-β-1a. All patients randomized in the PRISMS study were eligible for enrolment into this long-term follow-up study, regardless of whether their participation in the original PRISMS study had been terminated. The long-term follow-up consisted of one scheduled assessment as close as possible to the seventh or eighth anniversary of PRISMS enrolment [24]. A lower proportion of patients initially treated with s.c. IFN-β-1a progressed by at least one point in their EDSS score compared with patients in the DT group: 73.3% for DT, 64.1% for 44 µg (p = 0.007 versus DT), and 67.7% for 22 µg (p = 0.036 versus DT). Patients who initiated treatment with s.c. IFN-β-1a had a lower relapse rate compared with patients in the DT group: 0.78 for DT, 0.60 for 44 µg (p = 0.014 versus DT), and 0.63 for 22 µg (p < 0.001). The T2 burden of disease was significantly lower in patients initially treated with s.c. IFN-β-1a 44 µg compared with patients who received DT: 24.5 for DT, 5.0 for 44 µg (p = 0.002) [24].

SPMS occurs during the later stages of MS and is characterized by a steady accumulation of disability, with or without superimposed relapses [25]. Most patients with RRMS will go on to develop SPMS [25]. In the Secondary Progressive Efficacy Clinical Trial of Recombinant IFN-β-1a in MS (SPECTRIMS), patients with clinically definite SPMS were randomized to s.c. IFN-β-1a (44 or 22 µg) or placebo t.i.w. for 3 years [26]. SPECTRIMS demonstrated that treatment with s.c. IFN-β-1a did not significantly affect disability progression at 3 years in patients with SPMS compared with those treated with placebo, suggesting that the study drug is less effective in patients with later-stage MS. However, patients treated with s.c. IFN-β-1a who had relapses in the 2 years prior to study start took longer to reach EDSS confirmed progression, compared with placebo, although this difference was not significant. These patients were significantly younger at baseline, had a shorter duration of disease, and had deteriorated faster than those in the non-relapsing group. A significant benefit in terms of relapse rate for both doses was observed at 3 years; relapse rates among patients with pre-study relapses were 0.67 for 44 µg and 0.57 for 22 µg versus 1.08 for placebo (p < 0.001 for each active group versus placebo) [26].

Further studies have explored different dosages and frequencies of IFN-β-1a treatment. The Once Weekly IFN for MS Study (OWIMS) compared the efficacy of s.c. IFN-β-1a 44 and 22 µg once weekly (q.w.) dosing in patients with MS versus placebo. Although both doses were significantly more effective than placebo, 44 µg was better than 22 µg at week 48 in terms of reducing T2 new lesions and burden of disease. However, low-dose s.c. IFN-β-1a demonstrated nonsignificant reduction in the median number of unique lesions at week 24 [27]. The results of OWIMS were inferior to those reported in the PRISMS study, thus supporting the s.c. IFN-β-1a t.i.w. regimen.

The Evidence of IFN Dose–response: European–North American Comparative Efficacy (EVIDENCE) study demonstrated that after 48 and 64 weeks of treatment, patients with MS were significantly less likely to relapse or have MRI activity if treated with s.c. IFN-β-1a 44 µg t.i.w., compared with i.m. IFN-β-1a 30 µg q.w. [28,29]. A 32-week extension of EVIDENCE established that switching from 30 µg q.w. (i.m.) to 44 µg t.i.w. (s.c.) significantly improved clinical and MRI outcomes (patients originally receiving 44 µg t.i.w. continued on this regimen), which was demonstrated by comparing outcomes at the end of the transition period to those before the transition period [30].

### 2.2 Efficacy of s.c. IFN-β-1a in patients with symptoms suggestive of MS

The first study to explore the effects of early treatment with s.c. IFN-β-1a was the Early Treatment in Multiple Sclerosis (ETOMS) study [7]. Patients enrolled had had a first episode suggestive of MS within 3 months of the start of the study and MRI evidence suggestive of MS. Patients were randomized to s.c. IFN-β-1a 22 µg or placebo q.w. for 2 years. ETOMS found that fewer patients converted to CDMS after 2 years when receiving s.c. IFN-β-1a, compared with placebo: 34 versus 45% (p = 0.047). It took 252 days for 30% of the placebo group to convert to CDMS, whereas in the treated group this proportion did not convert until day 569 (p = 0.023), representing a delay of 317 days. Patients receiving s.c. IFN-β-1a also had significantly fewer new T2 lesions at 2 years, compared with placebo (p < 0.001), and less brain atrophy. In both the s.c. IFN-β-1a and placebo groups, most patients met the requirements for MRI conversion (defined by the appearance of at least one new MRI lesion of > 10 mm or three new lesions < 10 mm). Despite this, the proportion of patients without MRI activity was significantly higher in the s.c. IFN-β-1a treatment group than in the placebo-treated group [7].

In contrast to the OWIMS study, where s.c. IFN-β-1a 22 µg was less effective compared with s.c. IFN-β-1a 44 µg in reducing T2 new lesions, burden of disease and unique lesions in patients with MS, s.c. IFN-β-1a 22 µg was effective in the ETOMS study. In ETOMS, patients were treated after an FCDE, demonstrating that earlier treatment yields a better response.

The promising results of the ETOMS study were followed by REFLEX – a multicenter Phase III study that aimed to determine the efficacy of s.c. IFN-β-1a in patients with an EDSS score < 1 who had displayed symptoms suggestive of MS within 60 days of study entry and who had at least two clinically silent T2 lesions on MRI of ≥ 3 mm on T2-weighted MRI scan [19]. Patients were randomized to s.c. IFN-β-1a 44 µg (t.i.w. or q.w.) or placebo for 24 months; however, on conversion to CDMS, patients were switched to s.c. IFN-β-1a 44 µg t.i.w. until the study end. To meet the 2005 McDonald criteria for MS, patients had to have evidence of dissemination of MRI lesions or a second clinical attack. The primary end point of REFLEX was time to a diagnosis of MS (2005 McDonald criteria).

Treatment after an FCDE with s.c. IFN-β-1a t.i.w. was shown to lower the risk of McDonald MS at 2 years, compared with s.c. IFN-β-1a q.w. and placebo (62.5% for t.i.w., p < 0.0001; 75.5% for q.w., p = 0.008; 85.8% for placebo) [19]. The cumulative incidence of conversion to CDMS at 2 years was lower for both s.c. IFN-β-1a regimens, compared with placebo (20.6% for t.i.w., p = 0.0004; 21.6% for q.w., p = 0.0023; 37.5% for placebo). MRI outcomes were also significantly improved in patients receiving s.c. IFN-β-1a, with the reduction in mean number of combined unique active lesions lower in those receiving the study drug (0.60 for t.i.w., p < 0.0001; 1.23 for q.w., p < 0.0001; 2.70 for placebo). Further, the mean number of combined unique active lesions (defined as a new or persisting gadolinium-enhancing lesion on T1 MRI or a new or enlarging lesion on T2 MRI that was non-gadolinium-enhancing on T1 MRI) per patient per scan was lower in the t.i.w. group, compared with the q.w. group (p = 0.0015).

In REFLEXION, the ongoing extension of the REFLEX study, patients originally receiving placebo who had not converted to CDMS at 2 years were switched to s.c. IFN-β-1a t.i.w. and those receiving the study drug continued on their original regimen if they had not converted to CDMS [20]. The effects seen in REFLEX patients who had received the study drug persisted for up to 36 months: the cumulative probability of CDMS was 27.1% (p = 0.002) for t.i.w. dosing, 27.6% (p = 0.006) for q.w. dosing and 41.3 for DT. A significant reduction in the mean change in T2 lesion volume and T1 hypointense lesion volume from baseline was also seen at 36 months [20,21]. Data from REFLEX and REFLEXION demonstrate that there is a significant benefit in treating MS soon after the FCDE. Patients who received DT had worse outcomes at 3 years in comparison with those who received early treatment.

## 3. Safety and tolerability

The PRISMS study provides one of the most complete long-term datasets (up to 8 years of safety outcomes) available for MS therapeutic options, providing s.c. IFN-β-1a with a well-established safety profile based on extensive clinical trial programs.

A long-term follow-up of patients from the PRISMS study, performed 7 – 8 years after PRISMS study start, demonstrated that s.c. IFN-β-1a was generally well tolerated, with no new safety concerns identified since the end of the PRISMS study. Safety was assessed by asking patients about any adverse events (AEs) that were present at the time of the long-term follow-up visit. The most frequently reported AE was application-site disorders, which were reported in 44.0% of patients receiving any IFN-β-1a, and the majority of AEs were classified as mild in severity (61.5%). Other IFN-β-1a-related AEs were reported at a much lower frequency, for example, flu-like symptoms were present in 11.7% of patients. Laboratory abnormalities were also reported in the PRISMS long-term follow-up study, although these were found to be infrequent and mild in severity. The most common liver abnormality was elevated alanine aminotransferase levels, affecting 8.4% of patients receiving s.c. IFN-β-1a, 20.0% of patients receiving other disease-modifying therapies (DMTs) and 7.6% of those receiving no DMT at long-term follow-up [24]. Such safety and tolerability is comparable with other s.c. IFN-β-1a studies [7,19,29].

Ongoing AEs, such as injection-site reactions and flu-like symptoms, have been found to decrease in frequency over time [9]. Flu-like symptoms may be managed using NSAIDs, whereas injection-site reactions may be managed with devices (e.g., automated devices with customizable settings), training or site rotation. Further, long-term follow-up has indicated that no new safety concerns with s.c. IFN-β-1a have been identified in 8 years after treatment initiation [24]. These data suggest that there are no (new) safety concerns with early initiation of s.c. IFN-β-1a.

## 4. Conclusion

**Figure 1.**
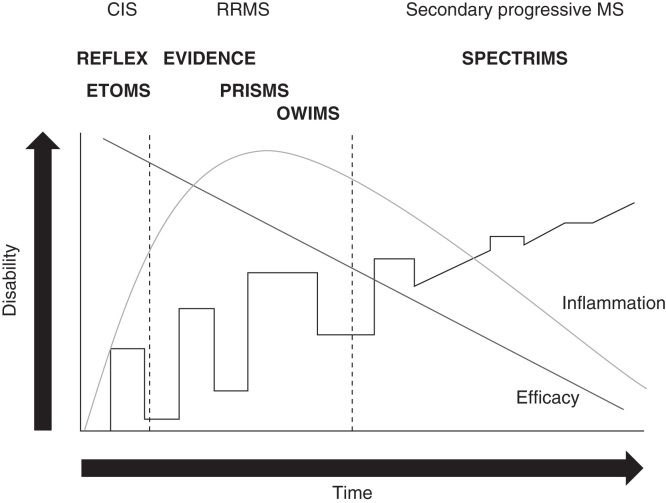
**Timeline of theoretical disease course of MS.** Response to s.c. IFN-β-1a diminishes when administered later in the disease course.

CIS: Clinically isolated syndrome; ETOMS: Early Treatment in Multiple Sclerosis; EVIDENCE: Evidence of IFN Dose–response: European–North American Comparative Efficacy; MS: Multiple sclerosis; OWIMS: Once Weekly IFN for MS Study; PRISMS: Prevention of Relapses and Disability by IFN-β-1a Subcutaneously in Multiple Sclerosis; s.c.: Subcutaneous; SPECTRIMS: Secondary Progressive Efficacy Clinical Trial of Recombinant IFN-β-1a in MS; REFLEX: Rebif Flexible Dosing in Early MS; RRMS: Relapsing–remitting multiple sclerosis.

Taken together, the data reviewed herein demonstrate that treatment with s.c. IFN-β-1a as early as possible in the course of the disease maximizes the response to therapy, both early and late. The studies discussed also indicate that high-dose, high-frequency administration of s.c. IFN-β-1a is more efficacious than low-dose, low-frequency administration. Data from REFLEX and REFLEXION highlight the beneficial effects of providing treatment after the FCDE, with patients receiving DT having significantly worse outcomes. These data support treatment initiation at the FCDE, confirming results reported for other IFN-β and GA trials [7-10]. Figure 1 shows the disease course over time of a typical case of MS, indicating how, while to some extent effective at all stages, s.c. IFN-β-1a is most effective in the early phases where inflammation predominates. REFLEXION is an ongoing study and will continue to follow patients for at least 5 years after the FCDE. This follow-up should allow clinicians to determine how early treatment translates to later effects on disease progression.

## 5. Expert opinion

Historically, the earliest possible time to start treatment was after a diagnosis of CDMS. Patients enrolled in the PRISMS study had a clinically definite or laboratory-supported definite diagnosis of MS of 5.3 (median) years’ duration prior to the study start, and patients receiving placebo had this untreated period extended for a further 2 years [22]. Due to improved diagnostic criteria and changes to drug licenses allowing treatment after the FCDE, patients today tend to be treated much earlier, both in clinical practice and in drug trials. The efficacy and tolerability of s.c. IFN-β-1a discussed in this review suggest that if treatment is administered after the FCDE, conversion to MS could be significantly delayed and disability progression might be postponed; however, the long-term benefits of early s.c. IFN-β-1a treatment are yet to be determined; it is hoped that ongoing research will address this. Hence, one could hypothesize that if patients from the PRISMS study had been treated after the FCDE, as in the REFLEX study, they may well have better overall outcomes today.

The REFLEX study demonstrated that early treatment (i.e., following the FCDE) with s.c. IFN-β-1a significantly reduces the risk of FCDE conversion to MS, whereas the ongoing REFLEXION study will establish the long-term effects of administration of s.c. IFN-β-1a following an FCDE, allowing us to determine whether the benefits currently seen with early treatment persist.

The rationale for early treatment of MS is supported by a study that assessed the differences between patients with MS receiving early versus late IFN-β treatment in terms of the risk of a 1-point progression in EDSS score and the ‘milestone’ EDSS scores of 4.0 and 6.0 [31]. The lowest risk was found to be when treatment was initiated within 1 year of MS onset, which significantly reduced the risk of a 1-point progression in EDSS score, compared with treatment initiated > 1 year from MS onset.

The development of neutralizing antibodies (NAbs) to IFN-β treatment has been known for some time. There is, however, no universally agreed methodology on how they should be measured or what titer may be important. The overall incidence of NAbs is low and they may be a transient phenomenon, making it difficult to demonstrate how their presence may change treatment decisions and outcomes. Physicians are reluctant to stop IFN in patients who are responding well to treatment, irrespective of NAb status, but in patients who are not responding well, IFN will be switched and finding a negative NAb titer will not change that decision. Although *in vitro* studies demonstrate evidence of NAb production with IFN-β treatment, the effect of NAbs on treatment efficacy has not been fully established. Indeed, the 5-year follow-up of the BENEFIT study, in which patients with an FCDE suggestive of MS were treated with s.c. IFN-β-1b and followed for 5 years, demonstrated that the presence of NAbs was not associated with a shorter time to CDMS or a higher annualized relapse rate [32].

The information reviewed herein underlines the importance of treating all patients at the early stage of the disease, where there is a window of opportunity to reduce CNS damage in the hope of reducing disability in the short and long term. With this growing knowledge, and the ultimate aim of the treatment of MS to inhibit irreversible axonal damage, it is important that all patients with an FCDE are considered for treatment as early as possible, even though we cannot be certain when they will convert to CDMS.

Article highlights.

- There is a wealth of evidence that suggests that multiple sclerosis (MS) should be treated as early as possible in the disease course, with patients receiving delayed treatment having significantly worse outcomes than those receiving early treatment.
- Continuous advances in MS diagnostic criteria allow early diagnosis and treatment to be initiated as soon as signs of MS appear.
- Subcutaneous IFN-β-1a is an effective medicine for treating early MS and is generally well tolerated.
- It remains to be determined whether there are long-term benefits of early treatment, although it is hoped that current ongoing research will answer this question.

This box summarizes key points contained in the article.

## Declaration of interest

MS Freedman (or his professional corporation) has received personal compensation from Actelion, Bayer HealthCare, Biogen Idec, EMD Serono (Canada), Genzyme, Novartis, Opexa, Sanofi-Aventis and Teva Canada Innovation. This review was funded by an independent medical grant from Merck Serono SA Geneva, Switzerland, a subsidiary of Merck KGaA, Darmstadt, Germany. The author has no other relevant affiliations or financial involvement with any organization or entity with a financial interest in or financial conflict with the subject matter or materials discussed in the manuscript apart from those disclosed.
